# Metrological and Algorithmic Traceability of Machine Learning in Laboratory Medicine

**DOI:** 10.1002/jcla.70321

**Published:** 2026-07-27

**Authors:** Qing Li, Mario Plebani

**Affiliations:** ^1^ Reference Measurement Laboratory Shanghai Center for Clinical Laboratory Shanghai China; ^2^ Department of Medicine‐DIMED University of Padova Padova Italy

**Keywords:** algorithmic traceability, artificial intelligence, harmonization, laboratory medicine, machine learning, metrological traceability, uncertainty quantification

## Abstract

**Background:**

The integration of machine learning (ML) into clinical laboratory medicine offers new opportunities for quality improvement. Models are now being developed to assist test interpretation, predict clinical outcomes, and support laboratory operations. However, these applications also require traceability that can accommodate both measurement science and data‐driven computation.

**Methods:**

We examine how metrological traceability relates to algorithmic traceability. We also examine three broad applications of ML in laboratory medicine and consider their different traceability requirements.

**Results:**

Metrological traceability links measurement results to reference systems through documented calibration hierarchies and the evaluation of uncertainty. Algorithmic traceability, by contrast, concerns whether the computational path from source data to model output can be reconstructed and audited. ML outputs therefore require careful interpretation according to their intended use. A model may use metrologically traceable laboratory results as inputs, yet the computational transformation that follows demands its own documentation, validation, and governance. Across the three broad applications, common problems persist. Laboratory data are not always comparable across methods, information leakage can be difficult to detect, uncertainty is frequently underreported, and model performance may shift after deployment.

**Conclusions:**

We propose a functional framework that aligns metrological principles with algorithmic documentation while preserving the distinction between reference materials and training datasets. A tiered approach to regulation and standards, supported by traceability dossiers and international collaboration, may help ensure that AI/ML systems in laboratory medicine are reproducible, comparable, and clinically fit for purpose.

## Graphical abstract

Machine learning is increasingly used in clinical laboratory medicine for test interpretation, outcome prediction, and laboratory operations, but its safe implementation requires traceability that covers both measurement science and data‐driven computation. Metrological traceability links laboratory results to reference systems through calibration hierarchies and uncertainty evaluation, whereas algorithmic traceability documents the data, transformations, model versions, validation, and monitoring needed to reconstruct model outputs. By distinguishing these two forms of traceability and applying intended‐use‐based validation, uncertainty‐aware reporting, and change control, AI/ML systems in laboratory medicine can become more reproducible, comparable, and clinically fit for purpose.

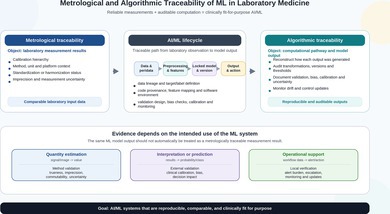

## 1 Introduction

In recent years, artificial intelligence (AI), particularly machine learning (ML), has generated substantial interest for applications in laboratory medicine [1]. ML methods are now capable of completing complex, well‐defined tasks with performance characteristics comparable to those of expert human operators [2]. Promising applications span the total testing process [3]. Some systems improve test requesting [4]. Others detect pre‐analytical and analytical errors [5, 6, 7, 8] or interpret complex multianalyte panels [9, 10, 11]. Prediction of disease onset or clinical deterioration represents another active area [12].

Despite this rapid growth, routine clinical implementation remains limited; most published ML approaches are still research prototypes rather than integrated tools within daily laboratory workflows [13]. This translational gap reflects the methodological and semantic complexity of laboratory data. A laboratory result is shaped by the method used to produce it. It carries the unit and calibration system of the measuring system, and it reflects the reagent lot and instrument platform on which it was generated. The pre‐analytical condition of the sample also matters, as do biological and analytical variability. These characteristics distinguish laboratory data from many other types of healthcare data and make traceability a central requirement for safe use of ML in this field.

Using ISO 17511 as a conceptual point of departure, this viewpoint discusses how traceability principles can be applied to ML in laboratory medicine. Metrological traceability is kept linked to measurement results and to the laboratory data used as model inputs, whereas algorithmic traceability describes the processes needed to make ML pipelines reproducible. These processes include documentation, validation, monitoring, and change control. Together, these perspectives provide complementary assurance for trustworthy development and deployment of AI/ML systems.

## 2 Metrological and Algorithmic Traceability: Different Objects, Complementary Aims

Metrological traceability is the property of a measurement result whereby the result can be related to a reference through a documented, unbroken chain of calibrations, each contributing to measurement uncertainty [14]. Its object is the value assigned to a calibrator, trueness control material, or human sample. Its purpose is to make measurement results comparable and reliable across different IVD medical devices and over time.

In this context, algorithmic traceability denotes the capacity to reconstruct, audit, and reproduce the computational pathway by which an AI/ML system generates an output. Instead of following a calibration chain, it follows the model lifecycle. It asks where the data and labels came from and how they were transformed. It records which code and model version were used. It documents what validation supported the intended use, and it tracks how later changes in performance or implementation are controlled. The term is less formalized in laboratory medicine than metrological traceability in ISO 17511. However, it refers to the practical need for ML development to be reproducible, auditable, and controlled across its lifecycle [13, 15, 16, 17].

The distinction matters because laboratory ML outputs differ in kind. When an ML model directly estimates a measurand from a signal or image, the output may need to be evaluated as a measurement result or as part of a measurement system. In contrast, many laboratory ML applications produce interpretive outputs based on existing laboratory results. These may take the form of probabilities, classifications, alerts, or decision support signals. In such cases, the input measurements may be metrologically traceable, while the model output itself requires separate documentation. This includes performance validation, communication of uncertainty, and post‐deployment monitoring, rather than a calibration hierarchy in the ISO 17511 sense.

## 3 Standardization and Harmonization of Laboratory Data

Standardization and harmonization are related but distinct concepts. Standardization is achievable when a higher‐order reference measurement system exists. This system comprises reference materials and reference measurement procedures that allow results to be linked to a common reference through metrological traceability. Harmonization is needed when such reference systems are incomplete or unavailable. In these cases, the practical goal is to improve agreement or clinical equivalence among methods, which may be achieved through consensus procedures, the use of commutable materials, or empirically derived relationships [18, 19, 20, 21].

For ML development, this distinction is operationally important. When standardized laboratory results are available, model development and validation should preserve the relevant traceability information. This includes the method and unit, the calibration route, the instrument and reagent context, and any available information on imprecision or uncertainty. When only harmonization is feasible, the degree and limits of comparability should be documented, and model performance should be assessed across the analytical methods and clinical settings in which deployment is intended. A model trained on one method or platform may not retain performance when transferred to another, even if the same analyte name and unit are used.

## 4 ML Use Cases in Laboratory Medicine

ML in laboratory medicine encompasses at least three use cases with different traceability implications. In the first, a system assigns or estimates a quantity from an analytical signal or instrument output. This category includes estimations from images, spectra, and other digital outputs. Here, metrological traceability may be relevant not only to the input signal but also to the assigned value, and validation may resemble that of a measurement procedure. In the second, a model uses existing laboratory results to support interpretation or prediction. Examples include multianalyte interpretation, risk prediction, and diagnostic classification. These models depend strongly on the quality and comparability of their input data, but their outputs are usually clinical predictions or classifications rather than metrologically traceable measurement results. In the third, ML supports laboratory operations. Typical examples include quality control, workflow optimization, and the detection of errors such as sample misidentification. These tools mainly require algorithmic traceability, local validation, and ongoing monitoring, although they may still rely on metrologically traceable inputs [4, 5, 6, 7, 8, 9, 10, 11, 12, 13, 15].

Distinguishing these use cases clarifies the type of evidence needed before implementation. Quantity‐estimation systems require evidence familiar from method validation. This includes alignment with the reference system, commutability assessment, and evaluation of trueness, imprecision, and measurement uncertainty. Predictive or interpretive systems require evidence that their predictions are clinically valid and externally validated. They should also be calibrated and assessed for bias and decision impact. Operational systems require evidence that they work in the local workflow, that false alerts and missed events are understood, and that procedures exist for override, escalation, and model updates.

## 5 ISO 17511 and Functional Correspondences with ML Pipelines

ISO 17511:2020 establishes requirements for metrological traceability of values assigned to calibrators, trueness control materials, and human samples in in vitro diagnostic medical devices [14]. The standard describes calibration hierarchies that may include higher‐order reference measurement procedures and reference materials, as well as manufacturer‐selected procedures and commercial calibrators, all the way down to end‐user measurement systems. Each step contributes uncertainty that must be considered when assigning values and interpreting patient results.

ML pipelines also require structured documentation. Their relationship to ISO 17511 concepts is best framed as functional correspondences rather than direct metrological equivalents. Expert consensus labels, clinical classifications, or outcomes used for model training are often necessary target definitions, but they are not automatically higher‐order reference standards. Likewise, curated datasets may be high quality and reproducible, but they are not certified reference materials and do not transfer assigned values through a calibration hierarchy. Table 1 therefore presents conceptual analogies that can guide documentation and governance while preserving the boundary between measurement traceability and algorithmic traceability.

**TABLE 1:** Functional correspondences between ISO 17511 metrology concepts and ML pipeline documentation.

Metrological concept	ML pipeline element or documentation object	Functional correspondence and limitation
Primary reference measurement procedure	Target definition, clinical endpoint, annotation protocol, or adjudication process	Defines what the model is intended to estimate, classify, or predict. It is not a primary reference measurement procedure unless the target value is itself assigned by a higher‐order measurement procedure
Reference materials, including CRMs when available	Curated development, validation, or benchmark datasets with documented provenance and quality checks	Provides a stable evidence base for training or evaluation. A dataset, even when carefully curated, is not a CRM and does not assign values through a calibration hierarchy
Manufacturer‐selected measurement procedures and working calibrators	Feature engineering, preprocessing, model training, and hyperparameter optimization	Establishes the input–output rule used by the algorithm. Optimization may improve fit but does not replace calibration traceability of measurement results
End‐user measurement systems	Locked AI/ML system with its software environment, data interfaces, workflow integration, monitoring plan, and local implementation protocol	Represents routine use in a defined setting. Local verification is required because performance can change with population, instruments, workflows, or data distributions
Systematic measurement error	Algorithmic bias, probability miscalibration, subgroup performance differences, or deployment drift	Identifies systematic deviation from the intended target or performance claim. Bias may arise from labels, sampling, analytical methods, clinical workflows, or model design
Calibration traceability chain	Data lineage, code provenance, model versioning, environment capture, and change‐control record	Allows reconstruction of how an output was generated. This is an audit trail for computation, not a chain of calibrations linking a measured value to a reference system
Measurement uncertainty budget	Predictive uncertainty, probability calibration, confidence or prediction intervals, and uncertainty‐aware reporting	Communicates limitations of model output. Appropriate methods depend on the task, validation data, and deployment context
External quality assessment and interlaboratory comparison	Independent external validation, site‐to‐site evaluation, robustness testing, and post‐deployment monitoring	Tests whether performance generalizes across settings. It supports confidence in intended use but does not create unrestricted generalizability

## 6 Algorithmic Traceability in the ML Lifecycle

### 6.1 Problem Formulation, Data Preparation, and Leakage Prevention

Algorithmic traceability begins with translating a clinical or operational scenario into a clearly specified ML task. Before model development, it should be clear what the system is meant to do and what information it will use. The timing of predictions, the target population, and the clinical setting must also be defined. The acceptable level of risk should be explicit. This step is especially important in laboratory medicine because analyte names may conceal substantial method‐dependent variability, and a model trained on data from one platform or workflow may not be valid in another.

Data selection must be biologically and clinically valid, and the model should be trained and evaluated on representative cases. A common threat to validity is data leakage, which occurs when information unavailable at the time of prediction enters the modelling process. Leakage can occur in obvious ways, such as using the test set during feature selection, but also in more subtle ways, such as estimating preprocessing parameters before the train‐test split or including future outcomes as predictors. Traceable ML development therefore requires clear separation of training, validation, and test data. The test set should remain untouched, and preprocessing or tuning should be reported transparently [1, 13, 15, 22].

### 6.2 Metadata, Peridata, and FAIR Principles

Accurate interpretation of laboratory variables requires contextual information. Metadata describe the structure and meaning of the data, including variables, units, coding systems, instruments, and methods. Peridata describe the circumstances around a laboratory observation, such as the sample, timing, analytical context, and patient‐related conditions that may affect interpretation [23]. For example, interpretation of an aspartate aminotransferase result may depend on whether the sample was hemolyzed and when it was collected. It may also depend on whether the patient was fasting, which method was used, and what reference interval applies.

Algorithmic traceability should therefore cover not only the variables themselves, but also how they were processed, derived, mapped to common terms, converted between units, or handled when missing. These requirements align with Findable, Accessible, Interoperable and Reusable (FAIR) principles. Where governance permits, data dictionaries and code should be findable and reusable. The same applies to model documentation and traceability dossiers, which should support interoperability as well as external review [16, 17, 23, 24].

### 6.3 Versioning, Change Control, and Monitoring

The clinical behavior of an AI/ML system depends on details around the trained model as much as on the model itself. Many elements can alter the output seen by the user. These include preprocessing code and feature definitions, data interfaces and thresholds, software dependencies, and the deployment environment. Each clinical release should therefore have a locked model version and a record of the data snapshot and dependencies used to build it. When a model is retrained, a threshold is changed, or an input mapping is modified, the performance claim should be reassessed against the intended use.

Post‐deployment monitoring is essential because laboratory data distributions can shift over time. Many factors may change, including reagent lots and analyzer platforms, population mix and clinical pathways, coding practices, and sampling procedures. Monitoring should not be limited to overall accuracy. It should check whether incoming data have changed, whether missingness or output rates are drifting, and whether probabilities remain calibrated. Practical warning signs include excessive alerts, frequent overrides, subgroup performance problems, and changes in downstream clinical outcomes [13, 15, 25, 26].

## 7 Core Challenges in ML‐Enabled Traceability

### 7.1 Model Transparency and Interpretability

The black‐box nature of many ML algorithms presents a challenge for clinical assurance. Traditional analytical methods can often be described by measurement equations and uncertainty components. In contrast, complex neural networks and ensemble methods may achieve high predictive accuracy while obscuring decision pathways. This opacity is particularly problematic when a model output influences diagnostic interpretation, triage, or follow‐up testing.

Explainability techniques, including SHAP (SHapley Additive exPlanations) and LIME (Local Interpretable Model‐agnostic Explanations), can help identify features that contributed to individual predictions. However, a local explanation is not the same as a complete understanding of model behavior, data dependence, or causal validity. Explainability should therefore be one part of a broader evidence package, alongside clear task definition, external validation, uncertainty quantification, drift monitoring, and clinical governance.

### 7.2 Uncertainty in ML‐Based Laboratory Applications

There is currently no single calculation framework for uncertainty in laboratory applications incorporating ML. For model evaluation, uncertainty is usually expressed as interval estimates around performance measures, and recent reporting and evaluation guidance emphasizes that these estimates should be reported with a clear description of the validation design and calculation method [27, 28, 29, 30]. Bias, calibration, and applicability should be assessed on independent data, ideally including external or temporally separated validation datasets [28, 29, 30, 31].

For individual model outputs, confidence intervals around overall model performance are not sufficient. Uncertainty may be expressed through model‐specific or model‐agnostic approaches, including probabilistic calibration, conformal prediction, Bayesian methods, or ensemble‐based methods [32, 33, 34, 35]. When laboratory measurement uncertainty needs to be propagated through an ML model, Monte Carlo simulation may be useful, particularly for nonlinear models [36]. The important point is not to report one unexplained uncertainty value, but to state what has been evaluated, how it was estimated, whether bias and calibration were assessed, and how the remaining uncertainty affects the intended clinical use.

### 7.3 Data Quality, Representativeness, and Generalizability

The quality of ML models is constrained by the quality of their training and validation data. Laboratory data present distinctive challenges. Results vary by method. Biological and pre‐analytical factors matter. Values may be missing or censored, units may differ, and instruments or reagents may change over time. The EFLM checklist for AI/ML studies emphasizes documentation of analytical methods, units, instrumentation, biological variability, preprocessing, and validation procedures [1]. ISO/IEC 5259 further highlights data quality characteristics relevant to analytics and ML [16, 17].

Dataset shift is a particular risk. A model trained on data from one analytical platform, institution, or patient population may perform poorly elsewhere, even when analytes are nominally standardized. External validation across independent sites and populations is therefore a requirement for broad claims of performance, not merely a desirable addition [22]. For local deployment, verification should evaluate whether the model remains valid for the local population, analytical methods, workflow, and intended clinical use.

## 8 Strategic Opportunities for Advancement

### 8.1 ML‐based Quality Control and Operational Support

ML‐based quality control systems can complement traditional internal quality control and patient‐based real‐time quality control approaches. By learning from historical data, such systems may detect problems that are difficult to capture with conventional statistical rules. These include analytical drift, sample misidentification, pre‐analytical errors, and workflow anomalies [5, 6, 7, 8]. These tools should still have a defined intended use and transparent input variables. They also require independent validation, monitoring of alert burden, and procedures for human review and escalation.

These operational applications may be suitable for proportionate implementation pathways because they often support laboratory professionals rather than replacing diagnostic judgment. Nevertheless, they can still affect patient care by triggering repeat testing, sample rejection, or workflow prioritization. Their validation should therefore include both technical performance and operational consequences [13, 15, 37].

### 8.2 Digital Traceability Dossiers

A practical mechanism for algorithmic traceability is a digital traceability dossier. The dossier would describe the intended use and clinical setting. It would also trace the development and validation data. Labels and features would be explained, and the model and software versions would be identified. Performance and uncertainty methods would be summarized, and records of validation, monitoring, and later changes would be kept.

The dossier should also explain how the laboratory‐specific characteristics of the input data was handled. At minimum, it should name the analyte and unit, the measurement procedure, and the platform. It should also record calibration status and reference interval, together with any available information on imprecision or uncertainty. It should further state whether results were standardized or only harmonized. The goal is to make the path from laboratory observation to model output auditable and reproducible within the limits of privacy and intellectual‐property constraints.

## 9 Regulatory and Standards Development Recommendations

### 9.1 Adapting Metrological Principles Without Overextending ISO 17511

ISO 17511 provides a foundation for measurement results, but the full lifecycle of ML in laboratory medicine extends beyond calibration hierarchies alone. For AI/ML systems that directly assign quantitative values or function as part of a measurement procedure, ISO 17511 principles may be highly relevant. For predictive, interpretive, or operational systems, assurance comes from a different evidence base. This encompasses trustworthy data provenance, well‐documented modelling, and appropriate validation. It also requires clear uncertainty communication, post‐deployment monitoring, and change control, together with attention to the metrological quality of laboratory inputs.

A tiered regulatory approach is appropriate. Low‐risk operational support tools may require local verification, documentation, and monitoring. By contrast, high‐risk systems that generate diagnostic interpretations, replace analytical steps, or influence major clinical decisions require stronger evidence. This usually means independent external validation, uncertainty reporting, and formal change control. Such proportionality would support innovation while maintaining the central laboratory principle that reported information must be fit for clinical purpose [13, 15, 25].

### 9.2 Assessment Standards and Repositories of Compliant Algorithms

Given the limited generalizability of many ML models, a universal certification pathway or warehouse for all algorithms would be difficult to implement. A useful standards approach would instead define the evidence expected for a stated intended use. Such evidence would address where the data and targets came from and how the data were prepared. It would cover how validation was designed and how performance and uncertainty were measured. It would also describe how bias and privacy risks were addressed, and how the model will be monitored and updated.

A curated registry or repository may be useful for algorithms intended for broad use, especially when models are locked, externally validated, and supported by transparent documentation. Such a registry should describe the intended use and compatible data types. It should also identify the analytical methods used for validation, the populations studied, and any known limitations. Monitoring expectations and update history should be included. It should not imply that a model is automatically valid in every laboratory. Local verification and post‐deployment monitoring would remain necessary, particularly when laboratory methods, populations, or workflows differ from those used for validation.

## 10 Limitations of the Proposed Framework

Several limitations should be considered. First, the relationship between ISO 17511 and ML pipelines is conceptual and functional, not a set of direct equivalences. Documentation alone does not convert training datasets, labels, or model outputs into metrologically traceable entities. These elements are not reference materials or reference measurement procedures in the traditional sense. Second, the framework is most straightforward for supervised learning, where inputs and targets can be explicitly defined. Other approaches require different emphases. For example, unsupervised or generative models have no simple target label. Their traceability rests more on data provenance and objective functions. It also depends on evaluation criteria, the constraints placed on the model, and the monitoring and human oversight used after deployment. Third, uncertainty quantification remains context dependent and may be poorly calibrated when validation data are small, biased, or not exchangeable with deployment data.

Fourth, generalizability is limited by differences in analytical platforms, populations, clinical workflows, and data semantics. External validation and local verification cannot be replaced by documentation alone. Fifth, algorithmic traceability does not solve broader questions of clinical utility. Issues such as ethics, fairness, liability, and user behaviour require separate attention. Finally, standards and regulatory infrastructure for ML in laboratory medicine remain developing. At this stage, the framework functions as a practical bridge between measurement science and ML governance, with future refinement expected as evidence, regulation, and standards evolve.

## 11 Conclusion

The integration of ML into laboratory medicine is not a departure from measurement science but an expansion of the evidence needed to support trustworthy laboratory information. Metrological traceability remains essential for measurement results and for laboratory data used as model inputs. Algorithmic traceability complements it by making the computational step inspectable: how a model was built, validated, monitored, and controlled after release.

A mature approach to ML in laboratory medicine should preserve the strengths of ISO 17511 while recognizing the distinctive nature of ML outputs. Functional correspondences between calibration hierarchies and ML pipelines can help identify what must be documented and governed, provided they are not interpreted as direct equivalences. Progress will require collaboration among laboratory professionals, data scientists, manufacturers, regulators, standards organizations, and healthcare institutions. Clear terminology is only the starting point. The same effort is needed in data and peridata handling, external validation, uncertainty‐aware reporting, and proportionate oversight. With these elements in place, AI/ML systems in laboratory medicine can become more reproducible, comparable, and clinically actionable across diverse healthcare settings.

## Conflicts of Interest

The authors declare no conflicts of interest.

## Data Availability

Data sharing not applicable to this article as no datasets were generated or analysed during the current study.
